# Outer membrane vesicles as versatile tools for therapeutic approaches

**DOI:** 10.1093/femsml/uqab006

**Published:** 2021-06-08

**Authors:** Franz G Zingl, Deborah R Leitner, Himadri B Thapa, Stefan Schild

**Affiliations:** Institute of Molecular Biosciences, University of Graz, Humboldtstrasse 50, 8010 Graz, Austria; Institute of Molecular Biosciences, University of Graz, Humboldtstrasse 50, 8010 Graz, Austria; Institute of Molecular Biosciences, University of Graz, Humboldtstrasse 50, 8010 Graz, Austria; Institute of Molecular Biosciences, University of Graz, Humboldtstrasse 50, 8010 Graz, Austria; BioTechMed-Graz, Austria; Field of Excellence BioHealth, University of Graz, 8010 Graz, Austria

**Keywords:** bacterial vesicles, immunomodulation, inflammation, host cell interaction, OMV, vaccine development

## Abstract

Budding of the bacterial surface results in the formation and secretion of outer membrane vesicles, which is a conserved phenomenon observed in Gram-negative bacteria. Recent studies highlight that these sphere-shaped facsimiles of the donor bacterium's surface with enclosed periplasmic content may serve multiple purposes for their host bacterium. These include inter- and intraspecies cell–cell communication, effector delivery to target cells and bacterial adaptation strategies. This review provides a concise overview of potential medical applications to exploit outer membrane vesicles for therapeutic approaches. Due to the fact that outer membrane vesicles resemble the surface of their donor cells, they represent interesting nonliving candidates for vaccine development. Furthermore, bacterial donor species can be genetically engineered to display various proteins and glycans of interest on the outer membrane vesicle surface or in their lumen. Outer membrane vesicles also possess valuable bioreactor features as they have the natural capacity to protect, stabilize and enhance the activity of luminal enzymes. Along these features, outer membrane vesicles not only might be suitable for biotechnological applications but may also enable cell-specific delivery of designed therapeutics as they are efficiently internalized by nonprofessional phagocytes. Finally, outer membrane vesicles are potent modulators of our immune system with pro- and anti-inflammatory properties. A deeper understanding of immunoregulatory effects provoked by different outer membrane vesicles is the basis for their possible future applications ranging from inflammation and immune response modulation to anticancer therapy.

## Teaser

This review covers diverse aspects of outer membrane vesicles as modulators of the innate immune response, as vaccine candidates and as effector delivery vehicles.

Outer membrane vesicles (OMVs), which typically range from 20 to 400 nm in diameter, are naturally secreted nonliving facsimiles of their donor cells. OMV production seems to be a common feature of all Gram-negative bacteria. Although several mutations cause reduced vesiculation levels compared with the parental wild-type strain (McBroom *et al*. 2006; Schwechheimer and Kuehn 2013; Schwechheimer, Kulp and Kuehn 2014), to our best knowledge no mutant with abolished OMV release has been reported so far. Thus, shedding of OMVs seems to be a beneficial feature of Gram-negative bacteria. Given the energy cost for replacing the surface and periplasmic components exported via the vesicles, it can be hypothesized that OMV formation provides an advantage for the donor cells and was therefore evolutionarily maintained. Indeed, multiple functions and involvement in diverse physiological processes have been reported for OMVs, including inter- and intraspecies communication, nutrient-scavenging mechanisms, biofilm formation, defense against phages and antimicrobial agents, pathogenesis, bacterial stress response and faster adaption to new environments (Nevot *et al*. 2006; Song *et al*. 2008; Kulkarni and Jagannadham 2014; Kulkarni, Nagaraj and Jagannadham 2015; Schwechheimer and Kuehn 2015; Reyes-Robles *et al*. 2018; Cooke *et al*. 2019; Zingl *et al*. 2020). In summary, OMVs seem suitable delivery vehicles for a variety of biomolecules. Currently, OMVs represent the only mechanism of Gram-negative bacteria for secretion and subsequent delivery of hydrophobic compounds to target cells at high concentrations. Moreover, experimental evidence indicates that luminal as well as surface-associated proteins of OMVs are fairly protected from proteases, extending the half-life of proteinaceous effectors in proteolytic environments (Kesty and Kuehn 2004). OMVs have been therefore suggested as a new secretion system type zero (Guerrero-Mandujano *et al*. 2017).

**Figure 1.**
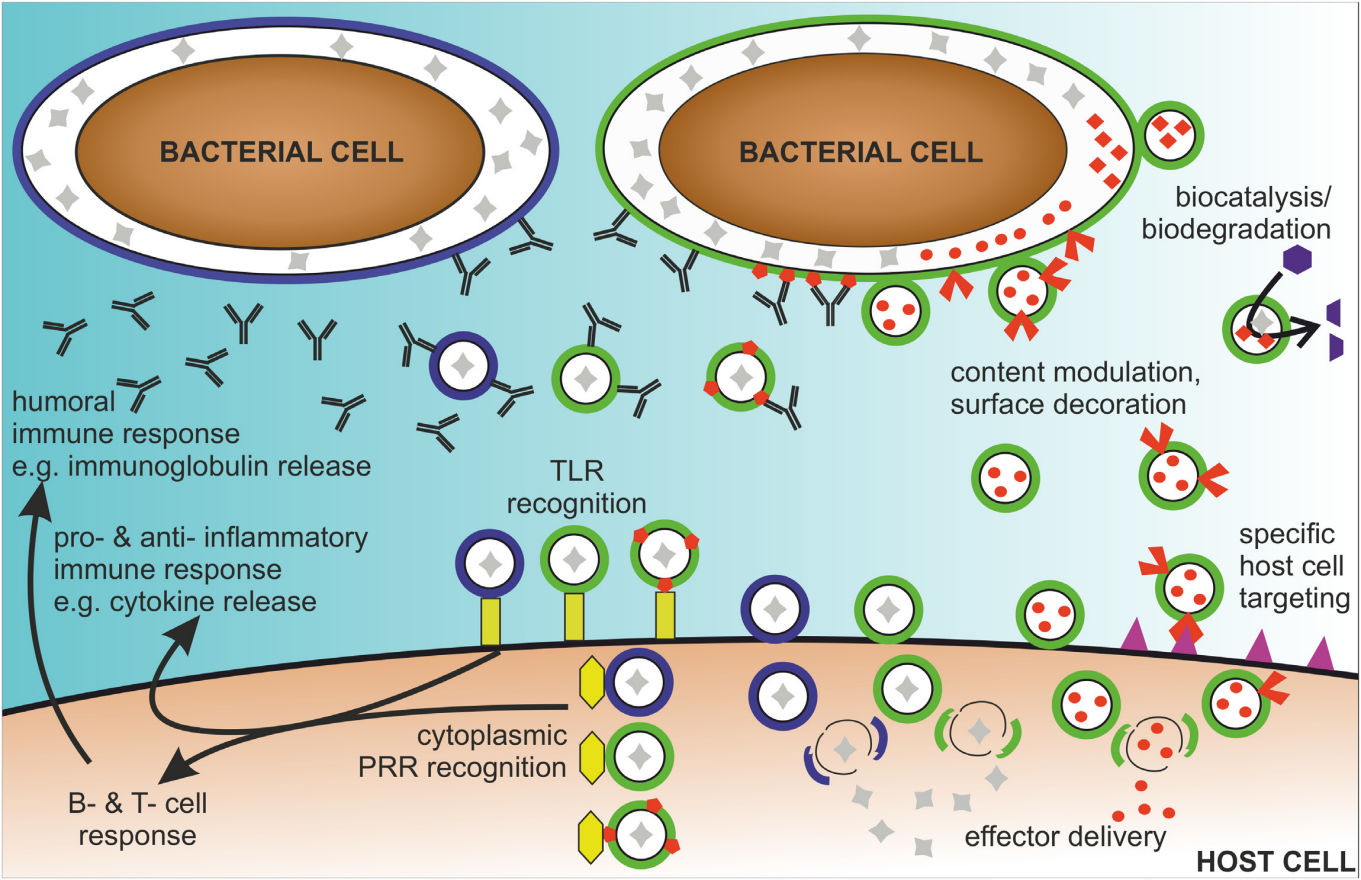
Overview of the therapeutic potential of OMVs. OMVs are released from bacterial cells (green or blue) with natural luminal content (gray). OMVs are recognized by host cell receptors (yellow), i.e. extracellularly via TLRs and in the cytosol after internalization via PRRs, resulting in inflammatory and humoral immune responses. Thus, OMVs could be used to specifically modulate pro- and anti-inflammatory responses. Moreover, OMVs can be used as vaccine candidates derived from an individual bacterial species (green or blue OMVs), derived from multiple species and used as mixtures (green and blue OMVs) or derived from genetically engineered to display heterologously expressed antigens (green OMVs with heterologously expressed antigens in red). Genetic engineering of donor bacterial cells also allows to modulate the composition of OMVs to decorate or load OMVs with heterologously expressed biomolecules (red components in or on OMVs). Applications of such bioengineered OMVs range from biocatalysis and biodegradation to delivery of natural and heterologously effectors to host cells, including targeting of specific host cell types via defined receptor interactions (purple).

Notably, OMVs are loaded with microbe-associated molecular patterns (MAMPs), such as lipopolysaccharides (LPSs), peptidoglycans, lipoproteins, proteins and nucleic acids, which are recognized by the immune system and allow them to interact with host cells and modulate the immune system. MAMPs on OMVs act as effectors, making them capable of manipulating the immune cascades in the host. This review focuses on three key features of OMVs offering potential therapeutical applications: (i) alteration of the host immune response, (ii) stimulating the immune system as potential vaccine candidates and (iii) delivery and/or protection of their cargo (Fig. 1).

## OMVS AS MODULATORS OF THE INNATE IMMUNE RESPONSE

OMVs can act as potent modulators of inflammatory responses in a wide range of host tissues, including the mucosal epithelial surfaces representing the first line of defense of our body (Fig. 1). The recognition of the OMV-associated MAMPs depends on specific host pattern recognition receptors (PRRs), which are present at the cell surface, in the endosomes or in the cytoplasm. Toll-like receptors (TLRs), nucleotide-binding oligomerization domain-like receptors, C-type lectin receptors and RIG-1-like receptors comprise the four families of PRRs. MAMP recognition by the host PRRs activates several host cell signaling cascades, thereby inducing expression of cell signaling molecules and antimicrobial peptides. Ismail and coworkers reported one of the first studies with regard to immunostimulatory capabilities of OMVs demonstrating a dose-dependent induction of a pro-inflammatory chemokine interleukin-8 (IL-8) upon stimulation of human gastric epithelial cells with OMVs derived from *Helicobacter pylori* (Ismail, Hampton and Keenan 2003). OMVs of *Neisseria gonorrhoeae*,*Pseudomonas aeruginosa* and *H. pylori* were shown to mediate a PRR response facilitating bacterial clearance and production of the antimicrobial peptide human β-defensin in human epithelial cells (Kaparakis *et al*. 2010). Moreover, several reports highlight the recognition of OMV-associated MAMPs by TLRs, representing membrane-spanning receptors with a cytoplasmic Toll/IL-1 receptor homology domain for downstream signaling (Jin and Lee 2008).

The activation of the TLRs is linked to the activation of transcription factors such as NF-κB (nuclear factor 'kappa-light-chain-enhancer' of activated B-cells) and MAP (mitogen-activated protein) kinase and to the release of pro-inflammatory cytokines, chemokines and type I interferons. For example, TLR3 and TLR7, which recognize dsRNA and ssRNA, have been shown to be stimulated by OMVs decorated with viral and parasite antigens (Fitzgerald and Kagan 2020; Yarovinsky *et al*. 2005). However, TLR4 is by far the most extensively studied member with regard to OMV signaling, as it interacts with lipid A, the hydrophobic membrane anchor and endotoxic part of the LPS (Poltorak *et al*. 1998; Hoshino *et al*. 1999; Park *et al*. 2009; Kawai and Akira 2010). OMVs derived from diverse bacteria, such as *Escherichia coli*, *P. aeruginosa* and *Fusobacterium nucleatum*, can activate the TLR4 cascade in various host cells resulting in activation of downstream NF-κB pathways and inflammatory cytokine release (Soderblom *et al*. 2005; Zhao *et al*. 2013; Engevik *et al*. 2021). Not surprisingly, several studies have shown that alteration of the lipid A moiety can massively alter the immune response. For example, OMVs containing underacylated LPS via deletion of lipid A acyltransferases, e.g. HtrB, MsbB or PagP, in donor strains, such as *Shigella*ssp., *Vibrio cholerae*, enterotoxigenic *E. coli* (ETEC) and *Salmonella*ssp., have been shown to reduce TLR4 stimulation and were therefore applied to OMV-based vaccine development to enhance tolerability (Rossi *et al*. 2014, 2016; Leitner *et al*. 2015). In case of the OMV-based *Neisseria meningitidis* vaccines, reduction of the LPS content was achieved by detergent extraction and resulted in less TLR4 activation (Fredriksen *et al*. 1991; Oster *et al*. 2005).

While TLR4 is essential for recognition of extracellular LPS, recent reports unraveled that intracellular cytosolic LPS can be recognized in a TLR4-independent manner via noncanonical caspase-dependent inflammasome activation (Hagar *et al*. 2013; Kayagaki *et al*. 2013). Importantly, OMVs are readily internalized by a variety of host cells and can function as transport vehicle for LPS (O'Donoghue and Krachler 2016). Indeed, OMVs derived from *E. coli* trigger cytosolic caspase activation resulting in pyroptosis and pro-inflammatory cytokine release (Vanaja *et al*. 2016). Moreover, OMVs of *Bordetella pertussis* have been recently shown to activate the noncanonical inflammasome pathway in murine macrophages, which is most likely triggered by the lipooligosaccharide component (Elizagaray *et al*. 2020). In contrast to the strict specificity of TLR4 for hexa-acylated lipid A, the caspase activation seems to be more relaxed allowing recognition of diverse lipid A variants with regard to the acylation pattern, but the precise molecular mechanism and structural requirements for the inflammatory caspase activation are not yet fully understood (Zamyatina and Heine 2020).

In addition, internalized OMVs can be recognized by cytoplasmic PRRs such as NOD1 and NOD2 resulting in phosphorylation of kinase signaling proteins, NF-κB activation and release of cell signaling proteins like cytokines and chemokines. These cytosolic receptors are recruited to the cytosolic compartment of the host cells after detection of bacterial peptidoglycan (Girardin *et al*. 2003). Peptidoglycans being a cell wall component of both Gram-positive and -negative bacteria are found to be enriched in OMVs. Studies performed with OMVs of *H. pylori*,*P. aeruginosa* and *N. gonorrhoeae* showed that the intracellular trafficking of peptidoglycan was essential for NOD1-mediated inflammatory response in nonphagocytic cells (Kaparakis *et al*. 2010). In addition, OMVs from *V. cholerae* non-O1 and non-O139, causing gastroenteritis and extraintestinal infections rather than cholera, have shown to elicit NOD1- and NOD2-dependent immune responses (Bielig *et al*. 2011). Apart from its sensory function, NOD receptor also contributes in maintaining intestinal homeostasis and microbiota balance (Rehman *et al*. 2011; Philpott *et al*. 2014). In this context, OMV-mediated stimulation of the NOD receptors can be seen as a mechanism by which the gut microbiota modulates the immune response.

Once OMVs have breached the epithelial barrier, they can also interact with various immune cells such as neutrophils, macrophages and dendritic cells in the host submucosa. For example, OMVs from *N. meningitidis* stimulate neutrophils resulting in the production of pro-inflammatory cytokines and chemokines [tumor necrosis factor alpha (TNF-α), IL-1β, IL-8, macrophage inflammatory proteins MIP-1α and MIP-1β] (Lapinet *et al*. 2000). On the contrary, OMV-mediated delivery of cytotoxic necrotizing factor type 1 (CNF1) toxin from uropathogenic *E. coli* impaired the antimicrobial and chemotaxis ability of neutrophils (Davis *et al*. 2006). OMVs are also recognized by human monocytes and macrophages resulting in the production of various pro-inflammatory cytokines and chemokines, e.g. reported for *N. meningitidis*,*H. pylori* or *Salmonella* ssp. (Alaniz *et al*. 2007; Tavano *et al*. 2009; Winter *et al*. 2014). Overall, the high abundance of MAMPs in OMVs can result in strong inflammatory responses by macrophages and monocytes, thereby exacerbating disease progression as shown for *Porphyromonas gingivalis* and *N. meningitidis* (Mirlashari *et al*. 2001; Ellis, Leiman and Kuehn 2010; Imayoshi, Cho and Kaminishi 2011). OMVs also interact with dendritic cells, which play a key role in the initiation of the primary immune response as they are the only antigen-presenting cell type capable of stimulating naïve T cells (Howard *et al*. 2004). OMVs from *N. meningitidis* induce the upregulation of MHC (major histocompatibility complex) class II molecules, costimulatory molecules such as CD40, CD80 and CD86, and the secretion of pro-inflammatory cytokines and chemokines in dendritic cells, resulting in a potent humoral immune response and maturation of dendritic cells (Durand *et al*. 2009). Additionally, OMVs can modulate immune cell signaling. For example, *N. meningitidis* OMVs were internalized by dendritic cells via binding to the host immune factor bactericidal permeability-increasing protein. The subsequent secretion of pro-inflammatory cytokines and induction of costimulatory molecules suggest a role of *N. meningitidis* OMVs in dendritic cell maturation (Schultz *et al*. 2007). Similarly, Cai *et al*. demonstrated that *Acinetobacter baumannii* OMVs activated bone marrow dendritic cells and promoted Th2 and humoral immune response (Cai *et al*. 2019). The potent immune cell stimulation by OMVs can be a beneficial feature along with their use as vaccine candidates providing adjuvant activity to enhance antibody and T-cell responses.

The cargo of OMVs, like endotoxins or RNA, is also able to modulate the host immune system. For example, the heat-labile enterotoxin (LT) from ETEC can be secreted in OMVs as well as in a free soluble form. Notably, activator protein 1 is essential for TNF-α and IL-6 activation by soluble LT, while OMV-associated LT induced TNF-α and IL-6 via an alternative pathway through NF-κB (Chutkan and Kuehn 2011). Furthermore, Vidakovics *et al*. showed that the OMVs derived from the respiratory pathogen *Moraxella catarrhalis* contain the *Moraxella* immunoglobulin D (IgD) binding protein (MID) and bacterial CpG-DNA motifs (Schaar *et al*. 2011). These OMVs induced a T-cell-independent B cell activation, via binding of MID to the IgD B cell receptor stimulating PRR signaling in B cells. In addition, MID-containing OMVs also activated lymphocytes in the tonsils resulting in IL-6 and IgM production (Vidakovics *et al*. 2010). In contrast, intracellular small RNAs (sRNAs) found in OMVs secreted by *P. aeruginosa* have been shown to downregulate the host immune response. The LPS-depleted sRNA-containing OMVs reduced IL-8 secretion in human airway epithelial cells and mouse lungs (Koeppen *et al*. 2016). The authors suggested that sRNA in OMVs might target cellular mRNA, thereby reducing IL-8 secretion, neutrophil recruitment and immune cells phagocyting bacteria (Koeppen *et al*. 2016). In another study, Choi *et al*. detected microRNA-sized small RNA (msRNA) in OMVs from *Aggregatibacter actinomycetemcomitans*,*P. gingivalis* and *Treponema denticola* (Choi *et al*. 2017). The OMV-associated msRNAs were able to suppress expression of several cytokines emphasizing the host–OMV interaction potential in periodontal disease (Choi *et al*. 2017).

Aside from their inflammatory properties, OMVs can also facilitate maintenance of gut homeostasis and activate antimicrobial defense mechanisms. For example, OMVs of *N. gonorrhoeae*, *P. aeruginosa* and *H. pylori* were shown to mediate a PRR response facilitating bacterial clearance and production of the antimicrobial peptide human β-defensin in human epithelial cells (Kaparakis *et al*. 2010). OMVs derived from *Bacteroides fragilis* and *Akkermansia muciniphila* induced anti-inflammatory responses and prevented gut inflammation in an experimental murine colitis model (Shen *et al*. 2012; Kang *et al*. 2013). In case of *B. fragilis* OMVs, the beneficial effect was pinpointed to the immunoregulatory capsular polysaccharide A, which is recognized by TLR2 in host dendritic cells promoting anti-inflammatory cytokine production and regulatory T-cell development (Shen *et al*. 2012). Moreover, OMVs from *A. muciniphila* also improved the intestinal barrier integrity in a high-fat diet mouse model outlining their role in the regulation of gut permeability (Chelakkot *et al*. 2018). *Escherichia coli* Nissle 1917 is probably the best-studied probiotic species among Gram-negative bacteria and has been associated with anti-inflammatory properties, epithelial barrier restauration, inhibition of epithelial invasion by pathogens and direct antagonistic effects to pathogenic bacteria (Sonnenborn 2016). It is becoming evident that OMVs derived from probiotic *E. coli* Nissle 1917 contribute to these beneficial features. For example, *E. coli* Nissle 1917 OMVs upregulate tight junction proteins (ZO-1 and claudin-14) strengthening the epithelial barrier integrity (Alvarez *et al*. 2016). OMVs from *E. coli* Nissle 1917 as well as the commensal ECOR12 also constantly activate NOD1 receptors, which suggests that OMVs derived from the gut microbiota trigger a controlled inflammatory response, which contributes to pathogen eradication and gut homeostasis (Canas *et al*. 2018). Moreover, OMV derived from the gut commensal *Bacteroides thetaiotaomicron* deliver BtMinpp, a bacterial homolog of eukaryotic multiple inositol polyphosphate phosphatase 1 (MINPP1), into host cells (Stentz *et al*. 2014). BtMinpp seems to have beneficial effects for both the host and the bacterial community by providing nutrients and by removing potentially carcinogenic metabolites from the gut (Stentz *et al*. 2014). These observations emphasize that OMVs can have a dual role of promoting inflammation and facilitating gut homeostasis.

## OMVS AS VACCINE CANDIDATES

The emergence of multidrug-resistant bacteria has highlighted the need to develop alternative strategies to combat bacterial infections. Among them are preventive measures like vaccines, which have the potential to reduce both the disease burden and the antibiotic use. A recent development of vaccine candidates included the use of OMVs. The fact that they closely resemble the surface of the donor bacterium make them interesting vaccine candidates. Although OMVs contain multiple surface-exposed antigens in their native confirmation, they are nonreplicating avoiding additional inactivation steps, which are applied to whole-cell killed vaccines. Hence, OMVs are highly immunogenic and combine native antigen presentation with proper adjuvant properties (Fig. 1). Moreover, genetic modification of the OMV-producing bacteria allows the improvement of the OMV yield as well as the enhancement of their immunostimulatory and safety profile (van der Pol, Stork and van der Ley 2015). As a result, OMVs have been studied as promising vaccine candidates for various diseases caused by Gram-negative pathogens (Micoli and MacLennan 2020).

Notably, vesicle formation is not restricted to Gram-negative bacteria. Thus, Gram-positive bacteria also release membrane vesicles, which have been studied as vaccine candidates. Among others, vesicles of *Clostridium perfringens*,*Streptococcus pneumoniae*,*Bacillus anthracis*,*Mycobacterium tuberculosis* and *Staphylococcus aureus* have been shown to elicit immune responses in mouse models and increase survival upon lethal challenge (Rivera *et al*. 2010; Jiang *et al*. 2014; Olaya-Abril *et al*. 2014; Athman *et al*. 2015; Choi *et al*. 2015; Wang *et al*. 2018). As this review focuses on OMVs derived from Gram-negative bacteria, we will not comprehensively discuss membrane vesicles of Gram-positive bacteria, but kindly refer to recent reviews on that subject for detailed information (Liu *et al*. 2018; Bose *et al*. 2020; Briaud and Carroll 2020; Caruana and Walper 2020).

Thus far, the most successful approaches are the OMV-based vaccines against *N. meningitidis*, which are licensed for human use and were successfully employed to control the meningococcal group B epidemic outbreaks in Cuba (VA-MENGOC-BC^®^, The Finlay Institute, Havana, Cuba), Norway (MenBVac^®^, Norwegian Institute of Public Health, Oslo, Norway) and New Zealand (MeNZB^®^, Chiron Vaccines, Auckland, New Zealand) (Holst *et al*. 2009, 2013). Furthermore, MenBVac was also successfully used to control an outbreak of *N. meningitidis* in the Normandy, France (Caron *et al*. 2011; Sevestre *et al*. 2017). To decrease endotoxin activity, LPS was removed from OMVs via detergent extraction, leaving the highly variable PorA protein as the immunodominant antigen (Pizza, Bekkat-Berkani and Rappuoli 2020). Although these vaccines were highly effective, they were only designed for protection against clonal outbreaks and the induction of a strain-specific immune response limited their broad application. To generate a vaccine against multiple meningococcal B strains, further research based on reverse vaccinology was utilized. This approach resulted in a multicomponent meningococcal B vaccine registered as Bexsero^®^ (Novartis). It combines three surface-exposed recombinant proteins added to OMVs from the epidemic outbreak in New Zealand and has already been approved for the market (Serruto *et al*. 2012).

Interestingly, recent epidemiological data including a retrospective case-control study have shown that OMV-based meningococcal vaccines can result in partial protection against gonococcal disease (Petousis-Harris *et al*. 2017; Petousis-Harris 2018). The observed cross-protection may be based on the high DNA homology of the two closely related species *N. meningitidis* and *N. gonorrhoeae* resulting in antibody cross-reactivity against meningococcal and gonococcal antigens (Tinsley and Nassif 1996). A recent study by Leduc and coworkers investigated this hypothesis in a well-characterized female mouse model of *N. gonorrhoeae* genital tract infection (Leduc *et al*. 2020). They demonstrated that vaccination of female mice with Bexsero (contains MeNZB OMVs) induces antibodies that recognize *N. gonorrhoeae* outer membrane proteins including several promising vaccine targets. This is in line with the study of Semchenko *et al*., wherein vaccination with Bexsero elicited antibodies to *N. gonorrhoeae* in rabbits and humans (Semchenko *et al*. 2019). These findings are consistent with the epidemiological data and suggest that a similar OMV-based vaccine could be a possible strategy to combat gonorrhea.

It should be mentioned that several other OMV-based vaccines are currently being developed for human and veterinary use. Strategies to combat *Salmonella enterica* infections in humans also include the elimination of the main source of infection, which is contaminated poultry products. A combination of a recombinant-produced *S. enterica* outer membrane protein F and extracted OMVs was shown to induce a strong antibody response in chicken and also rapidly decreased the bacterial load of the animals (Li *et al*. 2020a).

OMVs have also been successfully decorated with antigens of viral pathogens, such as the influenza A virus (H1N1) and MERS-CoV, which could extend the application of OMV-based vaccines to combat viral infections (Shehata *et al*. 2019). Recently, a OMV-based vaccine candidate against SARS-CoV-2 using OMVs from *N. meningitidis* as platform has been reported to induce a strong immune response in mice (Gaspar, Prudencio and De Gaspari 2021). Moreover, OMV-based vaccines against SARS-CoV-2 and Lyme disease are currently tested in preclincal trials (Intravacc 2021a,b).

As previously mentioned, LPS is a potent activator of TLR4, which contributes to vaccine reactogenicity. Thus, the reduction of the endotoxicity of an OMV-based vaccine is essential for a safe application in humans. In the case of the abovementioned MenB vaccines, this has been achieved by an additional detergent extraction step, which removes most of the LPS content but promotes aggregation (van de Waterbeemd *et al*. 2010). Furthermore, this approach alters the OMV composition through contamination of cytoplasmic proteins as well as removal of other membrane-located components, which may contribute to the OMV vaccines’ adjuvant and immunogenic profile (van de Waterbeemd *et al*. 2010, 2013a; Zariri *et al*. 2016). Thus, the absence of numerous potential antigens may require the use of additional adjuvants (Zariri *et al*. 2016; Gnopo *et al*. 2017). Due to these drawbacks, further research focused on the use of *N. meningitidis* strains with genetically modified LPS. These studies demonstrated that deletion of the lipid A acyltransferases, i.e.*LpxL1* and *LpxL2*, resulted in less endotoxic OMVs (van der Ley *et al*. 2001). Safety and immunogenicity of genetically detoxified *N. meningitidis* OMVs have been successfully assessed in phase 1 studies (Keiser *et al*. 2010, 2011).

Several studies have tried to optimize and standardize the manufacturing process of OMVs. One example is the production process developed for a *Shigella sonnei* OMV-based vaccine based on two filtration steps and genetically modified donor strains to increase the yield (deletion of *tolR*), removal of nicotinic acid auxotrophy (insertion of *E. coli nadAB* in *virG*) and reduction of endotoxicity (deletion of *htrB*) (Gerke *et al*. 2015). As such modifications are widely applicable to many Gram-negative bacteria that have been named ‘Generalized Modules for Membrane Antigens’ (GMMA) (Berlanda Scorza *et al*. 2012). Overall, this technology resulted in a GMMA-based *S. sonnei* vaccine candidate 1790GAHB, which has been successfully tested in animal and human trails (Gerke *et al*. 2015; Launay *et al*. 2017). Intramuscular administration of the vaccine candidate in healthy adult volunteers was well tolerated and induced antibody production against the O-antigen of *S. sonnei*, representing the dominant protective antigen (Launay *et al*. 2017). The vaccine candidate was also tested in a phase 2a clinical study in Kenya, a *Shigella*-endemic country, and was well tolerated with high immunogenic potency in African adults (Obiero *et al*. 2017). These promising results highlight the potential of genetically detoxified OMVs as vaccine candidates to combat bacterial diseases.

Aside from spontaneously released natural OMVs, bacterial vesicles can also be generated by cell lysis, e.g. using osmotic shock, physical shearing or detergents. As the yield of natural OMV production is considered to be a bottleneck along the upscaling process for vaccine production, it might be relevant to compare the protective efficacy of differentially produced vesicles. Using *A. baumannii* as model organism, Li and coworkers compared the yield, composition and protective properties of naturally released OMVs as well as vesicles induced by mechanical shearing or sucrose extraction (Li *et al*. 2020c). Interestingly, sucrose-extracted OMVs induced the highest protective immunity, which indicates the potential for alternative vesicle preparation strategies with increased yield and high protective efficacy for other OMV-based vaccine candidates. Besides higher yield, treatment of bacterial cultures with sublethal amounts of antimicrobial peptides can result in bacterial vesicles with reduced pro-inflammatory potency. Balhuizen and coworkers could show that sub-bactericidal concentrations of the cathelicidin PMAP-36 not only increase OMV production in *Bordetella bronchiseptica* but also are enclosed in the released OMVs and thereby attenuate undesired inflammatory responses, most likely via LPS neutralization (Balhuizen *et al*. 2021).

Mixtures of OMVs derived from multiple strains or even various organisms can be a promising candidate for a broad-spectrum vaccine. This approach has been already exploited in several studies. For example, Tunheim and coworkers reported that a combination of OMVs derived from *N. meningitidis* serogroups A and W induced high levels of functional antibodies against both serogroups in mice (Tunheim *et al*. 2013). Notably, the OMV-induced antibody titer was higher or equal to the levels induced by licensed meningococcal conjugate or polysaccharide vaccines (Tunheim *et al*. 2013). In line with that, Mitra and coworkers used OMVs derived from six *Shigella* strains to produce a multiserotype OMV vaccine against shigellosis (Mitra, Chakrabarti and Koley 2013). They demonstrated that immunization of adult female mice with the hexavalent OMV vaccine exhibited a consistent broad-spectrum antibody response, which protected the offspring passively against all four serogroups of *Shigella* (Mitra, Chakrabarti and Koley 2013). As OMVs consist of multiple antigens, a mixture of several strains is not always necessary as shown by a study introducing OMVs as vaccine candidates against nontypeable *Haemophilus influenzae* (NTHi) (Roier *et al*. 2012). Key limitations for vaccine development are the high genetic heterogeneity of NTHi strains and the enormous antigenic variability of several surface-exposed antigens (van Alphen *et al*. 1997; Erwin and Smith 2007). Interestingly, the induction of a protective immune response against several diverse NTHi strains was comparable regardless of whether only one OMV type or an OMV mixture derived from various heterologous strains was used for immunization (Roier *et al*. 2012). Most likely, the diverse antigens present in the OMVs are already sufficient to overcome the surface variability of NTHi strains.

This is in contrast to *V. cholerae*, where the O-antigen was identified as the major protective antigen of an OMV-based cholera vaccine (Leitner *et al*. 2013). This outermost compartment of the LPS is highly variable between different serogroups. Consequently, only a mixture of OMVs derived from *V. cholerae* serogroups O1 and O139 induced a high-titer protective immune response against both clinically relevant serogroups (Bishop *et al*. 2010). Leitner and coworkers extended this vaccine concept and demonstrated that immunization with a mixture of OMVs derived from ETEC and *V. cholerae* conferred protection against both pathogens (Leitner *et al*. 2015). Moreover, the study introduced genetically modified strains lacking not only a secondary LPS-acyl transferase but also the potent enterotoxins, i.e. cholera toxin or LT. Although the OMVs from these genetically modified strains exhibited less endotoxicity, they retained their potential to induce a high-titer, protective immune response.

As most reports simply mixed different OMVs in equal amounts, comprehensive dose response studies with variations in the OMV ratios are still lacking. Future initiatives need to address whether the efficacy can be improved upon optimization of the individual OMV amounts in immunization mixture and assess the maximum OMV diversity. This could establish well-balanced OMV combinations as a promising tool for the development of broadly protective vaccines against several Gram-negative pathogens.

Based on the natural traits of OMVs as delivery vehicles (see the next section), OMVs derived from genetically engineerable species have been considered as presentation platform for various heterologous antigens. These antigens need to be either deposited in the lumen of OMVs or displayed on the OMV surface. The target protein-based antigens are therefore fused to toxins (e.g. ClyA), outer membrane proteins (e.g. OmpA) or coupled to endogenous autotransporters (e.g. Hbp, AIDA) (Schroeder and Aebischer 2009; Chen *et al*. 2010; Bartolini *et al*. 2013; Daleke-Schermerhorn *et al*. 2014; Fantappie *et al*. 2014; Kuipers *et al*. 2015). Besides, the use of *E. coli* as host for OMV production also other species have been recently investigated, including *V. cholerae* for expressing ETEC antigens (CFA/I and Flic), *N. meningitidis* for expressing a borrelial surface-exposed lipoprotein (OspA) and *S. enterica* serovar Typhimurium for expressing the pneumococcal protein PspA (Muralinath *et al*. 2011; Leitner *et al*. 2015; Salverda *et al*. 2016). Furthermore, antiviral proteins can be displayed at the surface of OMVs exemplified by a study from Rappazzo and coworkers demonstrating that decoration of *E. coli* OMVs with M2e (ectodomain of an integral membrane protein of influenza A virus) conferred protection against influenza A infection in mice (Rappazzo *et al*. 2016).

As highlighted above for *V. cholerae*, protective antigens are not always proteins, but can also be glycan structures. Thus, OMVs have been investigated as carrier system for heterologous expressed glycan antigens. Using this approach, Chen *et al*. produced glycosylated OMVs that conferred protection against *Francisella tularensis* by expression of recombinant O-antigen on *E. coli* OMVs (Chen *et al*. 2016). Other glycosylated OMVs have been tested against *S. pneumoniae* in mice and against *Campylobacter jejuni* in chicken (Price *et al*. 2016). Furthermore, glycoengineered OMVs might open the possibility to target clinically important host glycans like those displayed on cancer cells. Proof of principle was reported by Valentine and coworkers demonstrating that immunization with glycosylated OMVs expressing the tumor-specific carbohydrate antigens polysialic acid and Thomsen–Friedenreich (T antigen) induced high titers of glycan-specific IgG antibodies (Valentine *et al*. 2016). In summary, these studies highlight the potential of OMVs as a vaccine platform for the development of polysaccharide vaccines against various glycan antigens of clinical relevance.

## OMVS AS EFFECTOR DELIVERY VEHICLES

Multiple Gram-negative bacteria have been shown to use OMVs as delivery vehicle for toxins, e.g. LT of ETEC, cholera toxin of *V. cholerae* or multiple toxins (Cif and PlcH) of *P. aeruginosa*, or to transport enzymes in an active conformation, e.g. proteases of *Lysobacter* sp. XL1 or *Pseudomonas fragi* (Thompson, Naidu and Pestka 1985; Wai, Takade and Amako 1995; Horstman and Kuehn 2002; Vasilyeva *et al*. 2008; Bomberger *et al*. 2009; Rasti, Schappert and Brown 2018). *Pseudomonas putida* is able to scavenge nutrients by loading several enzymes to catabolize aromatic compounds (Salvachua *et al*. 2020). Although OMVs reflect the periplasmic content and surface composition of the donor bacterium, several recent reports highlight enrichment or exclusion of defined components indicating cargo selection (Bonnington and Kuehn 2014). *Porphyromonas gingivalis* alters its protein cargo depending on the loading with proteases or LPS composition (Haurat *et al*. 2011). *Salmonella enterica* serovar Typhimurium exhibits differential composition of LPS in their OMVs upon shifting bacteria to a different pH condition, which promotes LPS remodeling of the outer membrane and might enhance bacterial fitness along environmental transitions (Bonnington and Kuehn 2016). Concordantly, a recent study demonstrated that enhanced OMV release by *V. cholerae* upon host entry allows a faster surface remodeling highlighted by the depletion of outer membrane porin OmpT and accumulation of glycine-modified LPS (Zingl *et al*. 2020). These surface adaptations confer resistance to host-derived antimicrobial agents and facilitate *in vivo* colonization fitness of the pathogen. *Helicobacter pylori* releases OMVs enriched with the protease HtrA, but lacking the type 4 secretion system component VirD4 (Olofsson *et al*. 2010). Comparative proteome analyses of OMVs and outer membrane derived from *N. meningitidis* revealed several distinct differences suggesting a selective packing of cargo into these vesicles (Lappann *et al*. 2013). For example, autotransporter family proteins, e.g. adhesins and proteases, as well as iron and zinc acquisition proteins were found to be enriched in OMVs, while outer membrane porins PorA and PorB as well as putative transglycosylases were drastically underrepresented in OMVs. Uropathogenic *E. coli* are able to specifically sort the CNF1 into OMVs, which seems to be dependent on the folate-binding protein YgfZ (Yu and Kim 2012). However, evidence for direct interaction between CNF1 and YgfZ is still lacking. Thus, the exact molecular mechanisms and bacterial strategies for differential sorting into the OMVs remain to be elucidated and will be valuable for future use of OMVs as vehicles for effector delivery.

OMVs have a natural ability to stabilize enzyme activity in their lumen and allow a flux of substrate and product through outer membrane porins. Thus, it is not surprising that multiple studies have been conducted investigating the beneficial effects of OMV encapsulation for enzymatic activity (Alves *et al*. 2015, 2016). Although these reports mainly focused on biocatalysis in biotechnological processes (Fig. 1), these initiatives can path the way for future therapeutical applications. Briefly, current studies highlight that temperature-sensitive enzymes protected within OMVs exhibit increased stability against repeated freeze/thaw cycles or prolonged storage at unfavorable, elevated temperatures (up to 37°C) than the soluble, purified enzyme version. Therefore, OMVs show the same advantages as conventional packing of enzymes into artificial lipid-based micelles or liposomes (Kraft *et al*. 2014), but can be purified by relative simple high-speed centrifugation, can be already preloaded by the bacteria and can enable the transport of hydrophobic compounds (Brameyer *et al*. 2018). The latter can be valuable for several biotechnological processes, since many enzymes of interest are highly unstable in aqueous solutions (Sada *et al*. 1987). For example, Alves and coworkers used OMVs enriched with a phosphotriesterase to efficiently degrade the organophosphate chemical warfare agent simulant paraoxon in environmental water samples highlighting the possible benefits of OMVs for bioremediation (Alves *et al*. 2018). Furthermore, OMVs decorated with organophosphorus hydrolases were used for biocatalytic decontamination of paraoxon, a highly toxic compound previously used as insecticide (Fu-Hsiang, Chih-Yun and Shen-Long 2017). The OMV-associated enzyme showed increased activity, prolonged half-life, improved temperature stability and enhanced pH tolerance (Fu-Hsiang, Chih-Yun and Shen-Long 2017).

To deposit heterologous proteins of interest in OMVs, several techniques have been reported (Li and Liu 2020b). A classical strategy comprises the fusion of the protein of interest to a signal sequence known to facilitate export to the periplasm. Upon accumulation in the periplasm, the protein of interest will be stochastically trapped in OMVs. Applicable secretion pathways include the Sec system or the twin arginine translocation (TAT) system (Oliver and Beckwith 1982; Chaddock *et al*. 1995). Latter has the advantage of exporting already folded proteins. Proof of principle was reported by efficient loading of *E. coli* OMVs with GFP (green fluorescent protein) fused to a TAT signal sequence, which allowed OMV detection by fluorescence measurements (Kesty and Kuehn 2004). Alternatively, the peptides of interest can be fused to target proteins, which are known to be transported to the outer membrane of the host bacterium. Prominent examples are fusions to the outer membrane protein OmpA or ClyA, which harbors a leader sequence resulting in the display of a functional protein on the surface of the vesicles (Kim *et al*. 2008, 2017a; Lee *et al*. 2009). Autotransporter systems, such as the *Neisseria* IgA protease or the *E. coli* AIDA-I, have also been successfully used to engineer strains for heterologous peptide display on OMVs, including ovalbumin fragments as well as *Leishmania* and ETEC antigens (Schroeder and Aebischer 2009; Hays *et al*. 2018; Schetters *et al*. 2019). A more broadly applicable system is the SpyTag/SpyCatcher system, which can form a covalent linkage between a protein of interest and the delivery system (Brune *et al*. 2016). Briefly, a protein of interest and an OMV-associated protein are tagged with either the SpyTag or the SpyCatcher domains, respectively. SpyTag and SpyCatcher domains interact with one another and form a covalent isopeptide bond. This technology has been recently used to link a phosphotriesterase to OmpA and thereby guiding the enzyme into OMVs (Alves *et al*. 2015). Using an ice nucleation protein anchoring motif, Park and coworkers managed to display a trivalent complex of functional cellulase domains on OMVs resulting in faster cellulose hydrolysis than the noncomplexed enzymes (Park *et al*. 2014). Similarly, the abovementioned organophosphorus hydrolases for pesticide degradation were displayed via the ice nucleation protein technology (Fu-Hsiang, Chih-Yun and Shen-Long 2017). OMV donor strains can also be genetically engineered to display glycan structures on their surface utilizing the loose glycan specificity of the O-antigen ligase. For example, the *S. pneumoniae* and *C. jejuni* surface glycans were expressed in an O-antigen *E. coli* variant resulting in glycosylation-modified OMVs, which induced a protective immune response in animal models (Price *et al*. 2016).

OMVs have been proposed to be used as a cell-specific drug delivery vehicle as they are able to target specific cells, can protect their cargo and are readily taken up by eukaryotic cells (Fig. 1). Interestingly, transmigration through epithelial cells and systemic distribution into distant organs, in particular the liver, was observed for OMVs derived from the gut commensal *B. thetaiotaomicron* upon oral administration (Jones *et al*. 2020). Thus, OMVs could be interesting candidates for long-distance, systemic distribution of effector molecules. Detoxified OMVs derived from *E. coli* K-12 Δ*msbB* have been shown to be effectively internalized by cancer cells via overexpression of an affibody on their surface (Gu *et al*. 2020). Affibodies were among the first biotechnological engineerable nonantibody scaffold molecules, which allow high-affinity binding to a large number of target proteins (Frejd and Kim 2017). They are small, robust proteins designed on the Z domain of the protein A from *S. aureus*. Affibodies imitate monoclonal antibodies and show similar antigen-binding capacity, yet are significantly smaller and exhibit improved stability properties. In the study of Gujrati and coworkers, they were used to target specifically a transmembrane receptor (HER2) overexpressed on several cancer cells (Gujrati *et al*. 2014). Loading of these vesicles with siRNAs to silence the expression of a kinesin spindle protein resulted in significant reduction of tumor growth in animal models (Gujrati *et al*. 2014). Similarly, detoxified OMVs derived from *E. coli* have recently been shown to deliver a tumor necrosis factor-related apoptosis-inducing ligand to cancer cells via integrin-targeting peptides and allow deep tissue penetration, which is important for tumor eradication (Gu *et al*. 2020). Notably, *E. coli* OMVs have already been shown to have natural tumor-suppressive properties by inducing the production of antitumor cytokines CXCL10 and interferon-γ (Kim *et al*. 2017b). These natural properties could be further amplified by presenting the ectodomain of programmed death 1 (PD1) on OMVs, which enabled the modified OMVs to bind the PD1 ligand 1 on tumor cells negating the inhibition of T-cell proliferation by tumor cells (Li *et al*. 2020d). Overall, the activity of the bioengineered OMVs, i.e. immune activation in the tumor microenvironment and suppression of immune evasion by the tumor cells, resulted in a marked reduction of tumor growth *in vivo* (Li *et al*. 2020d). Moreover, a recent study demonstrated that *Klebsiella pneumoniae* OMVs can be effectively enriched with the antitumor agent doxorubicin to treat lung cancer in the murine model (Kuerban *et al*. 2020). Notably, high cardiac toxicity, being a major adverse, dose-limiting effect of free doxorubicin, was significantly reduced upon association with OMVs (Kuerban *et al*. 2020).

Recent approaches also include the decoration of OMVs with tumor-specific epitopes as potentially tools for cancer immunotherapy. For example, Grandi and coworkers demonstrated that immunization of mice with *E. coli* OMVs enriched with epitopes of the tumor markers B16-M30 and B16-M30 was protected from a tumor challenge using B16F10EGFRvIII cells expressing the tumor markers, respectively (Grandi *et al*. 2017). Similarly, injection of *E. coli* OMVs enriched with tumoral antigen human papillomavirus type 16 early protein E7 induced potent antitumor CD4^+^ and CD8^+^ cell responses suppressing TC-1 tumor growth in mice (Wang *et al*. 2017).

Notably, the potent self-adjuvant properties of OMVs resulting in strong immunogenicity can be attributed to the high abundance of MAMPs on the bacterial surface, which trigger pro-inflammatory pathways (Mahla *et al*. 2013). A fine balance between immunogenicity and reactogenicity is key for successful treatment approaches of OMV-based drugs. While a refined triggering of inflammatory cascades can be beneficial for the humoral immune response to an OMV-based vaccine, a strong activation of innate immune responses may cause severe adverse effects, such as fever and septic shock. Such disadvantageous reactogenicity of OMV-based drugs needs to be carefully assessed and potentially minimized, especially in case of potential applications in immunocompromised patients. One of the most abundant and highly reactogenic components in OMVs is the LPS. As described above, specific extraction steps as well as genetic modifications of the donor bacterium can be applied to reduce the reactogenicity of the LPS in OMVs, while retaining their immunogenicity (Holst *et al*. 2009, 2013; Leitner *et al*. 2013; Mitra, Chakrabarti and Koley 2013). Practicability and potential requirement of further modifications in the context of immunocompromised patients remain to be elucidated. Aside from the high reactogenicity of OMV-associated MAMPs, additional challenges have to be addressed along the development of OMV-based cancer vaccines, including the selection of suitable antigens for the diverse cancer types, efficacy of heterologous antigen expression and removal of natural immunodominant or immunosuppressive components from OMVs (Zhang *et al*. 2019).

## CONCLUDING REMARKS AND FUTURE PERSPECTIVES

It is becoming increasingly evident that OMVs may be valuable tools for a variety of future medical applications, such as vaccination, cancer therapy and drug delivery. Most progress has been made along the vaccine development with first OMV-based vaccine being approved and commercially available. Thus, studies dealing with safety aspects and ‘good manufacturing practice’ production have been mainly focusing on this application (Frasch *et al*. 2001; van de Waterbeemd *et al*. 2010, 2013b; Berlanda Scorza *et al*. 2012; Rossi, Citiulo and Mancini 2021). As OMVs are produced by living bacteria, several steps are necessary to ensure safety, such as (i) close monitoring of bacterial growth, (ii) standardized isolation techniques using centrifugation, filtration and/or detergent extraction, (iii) sterilization and DNA removal, (iv) purity, quantity and composition analyses of the isolated OMVs and, if applicable, (v) the control of heterologous antigen expression. Furthermore, several assays have been described to assess reactogenicity and toxicity of isolated OMVs, such as the rabbit pyrogenicity test, the monocyte activation test, the limulus amoebocyte lysate test or receptor-specific tests in engineered cell lines (Rossi, Citiulo and Mancini 2021). OMVs are vital players for the bacteria–host crosstalk, e.g. via modulation and shaping of the host's immune response. OMVs may also be attractive multifunctional delivery vehicles for proteins, glycans or small-molecule drugs, which are protected and stabilized in association with OMVs. Moreover, OMVs are efficiently internalized by host cells and first reports indicate that OMVs can be used to target specific cell types. Understanding the host cell responses evoked by individual OMVs from diverse bacterial species, elucidating the principles of OMV internalization into target cells, identification of the involved bacterial effectors, and characterization of their cellular binding partners and pathways are necessary future steps for potential therapeutic applications. Furthermore, future research needs to address current limitations such as safety of OMVs, OMV loading or decoration with heterologous substances, and upscaling of OMV production and purification.

## ACKNOWLEDGMENTS

Apologies to all scientists studying OMVs whose work and research could be not mentioned in this review.

## FUNDING

This work was supported by the Austrian FWF grant P33073 to SS, DOC-50 (docfund ‘Molecular Metabolism’) to SS and W901-B12 (DK Molecular Enzymology) to FGZ and SS, by the Else Kröner-Fresenius-Stiftung, by BioTechMed-Graz, and by the Land Steiermark and City of Graz.

## References

[bib1] Alaniz RC , Deatherage BL, Lara JC et al. Membrane vesicles are immunogenic facsimiles of *Salmonella typhimurium* that potently activate dendritic cells, prime B and T cell responses, and stimulate protective immunity *in vivo*. J Immunol. 2007;179:7692–701. 18025215 10.4049/jimmunol.179.11.7692

[bib2] Alvarez CS , Badia J, Bosch M et al. Outer membrane vesicles and soluble factors released by probiotic *Escherichia coli* Nissle 1917 and commensal ECOR63 enhance barrier function by regulating expression of tight junction proteins in intestinal epithelial cells. Front Microbiol. 2016;7:1981. 28018313 10.3389/fmicb.2016.01981 PMC5156689

[bib4] Alves NJ , Turner KB, Daniele MA et al. Bacterial nanobioreactors: directing enzyme packaging into bacterial outer membrane vesicles. ACS Appl Mater Interfaces. 2015;7:24963–72. 26479678 10.1021/acsami.5b08811

[bib3] Alves NJ , Moore M, Johnson BJ et al. Environmental decontamination of a chemical warfare simulant utilizing a membrane vesicle-encapsulated phosphotriesterase. ACS Appl Mater Interfaces. 2018;10:15712–9. 29672020 10.1021/acsami.8b02717

[bib5] Alves NJ , Turner KB, Medintz IL et al. Protecting enzymatic function through directed packaging into bacterial outer membrane vesicles. Sci Rep. 2016;6:24866. 27117743 10.1038/srep24866 PMC4846811

[bib6] Athman JJ , Wang Y, McDonald DJ et al. Bacterial membrane vesicles mediate the release of *Mycobacterium tuberculosis* lipoglycans and lipoproteins from infected macrophages. J Immunol. 2015;195:1044–53. 26109643 10.4049/jimmunol.1402894 PMC4506856

[bib7] Balhuizen MD , Versluis CM, van Harten RM et al. PMAP-36 reduces the innate immune response induced by *Bordetella bronchiseptica*-derived outer membrane vesicles. Curr Res Microb Sci. 2021;2:100010. 34841304 10.1016/j.crmicr.2020.100010 PMC8610334

[bib8] Bartolini E , Ianni E, Frigimelica E et al. Recombinant outer membrane vesicles carrying *Chlamydia muridarum* HtrA induce antibodies that neutralize chlamydial infection *in vitro*. J Extracell Vesicles. 2013;2:20181. 10.3402/jev.v2i0.20181 PMC3760637 24009891

[bib9] Berlanda Scorza F , Colucci AM, Maggiore L et al. High yield production process for *Shigella*outer membrane particles. PLoS One. 2012;7:e35616. 22701551 10.1371/journal.pone.0035616 PMC3368891

[bib10] Bielig H , Rompikuntal PK, Dongre M et al. NOD-like receptor activation by outer membrane vesicles from *Vibrio cholerae* non-O1 non-O139 strains is modulated by the quorum-sensing regulator HapR. Infect Immun. 2011;79:1418–27. 21263023 10.1128/IAI.00754-10 PMC3067550

[bib11] Bishop AL , Schild S, Patimalla B et al. Mucosal immunization with *Vibrio cholerae* outer membrane vesicles provides maternal protection mediated by antilipopolysaccharide antibodies that inhibit bacterial motility. Infect Immun. 2010;78:4402–20. 20679439 10.1128/IAI.00398-10 PMC2950341

[bib12] Bomberger JM , Maceachran DP, Coutermarsh BA et al. Long-distance delivery of bacterial virulence factors by *Pseudomonas aeruginosa* outer membrane vesicles. PLoS Pathog. 2009;5:e1000382. 19360133 10.1371/journal.ppat.1000382 PMC2661024

[bib14] Bonnington KE , Kuehn MJ. Outer membrane vesicle production facilitates LPS remodeling and outer membrane maintenance in *Salmonella*during environmental transitions. mBio. 2016;7:e01532–16. 27795394 10.1128/mBio.01532-16 PMC5082901

[bib13] Bonnington KE , Kuehn MJ. Protein selection and export via outer membrane vesicles. Biochim Biophys Acta. 2014;1843:1612–9. 24370777 10.1016/j.bbamcr.2013.12.011 PMC4317292

[bib15] Bose S , Aggarwal S, Singh DV et al. Extracellular vesicles: an emerging platform in Gram-positive bacteria. Microb Cell. 2020;7:312–22. 33335921 10.15698/mic2020.12.737 PMC7713254

[bib16] Brameyer S , Plener L, Muller A et al. Outer membrane vesicles facilitate trafficking of the hydrophobic signaling molecule CAI-1 between *Vibrio harveyi* cells. J Bacteriol. 2018;200:e00740–17. 29555694 10.1128/JB.00740-17 PMC6040191

[bib17] Briaud P , Carroll RK. Extracellular vesicle biogenesis and functions in Gram-positive bacteria. Infect Immun. 2020;88:e00433–20. 32989035 10.1128/IAI.00433-20 PMC7671900

[bib18] Brune KD , Leneghan DB, Brian IJ et al. Plug-and-display: decoration of virus-like particles via isopeptide bonds for modular immunization. Sci Rep. 2016;6:19234. 26781591 10.1038/srep19234 PMC4725971

[bib19] Cai W , Kesavan DK, Cheng J et al. Vesicle-mediated dendritic cell activation in *Acinetobacter baumannii* clinical isolate, which contributes to Th2 response. J Immunol Res. 2019;2019:1. 10.1155/2019/2835256 PMC7012244 32083139

[bib20] Canas MA , Fabrega MJ, Gimenez R et al. Outer membrane vesicles from probiotic and commensal *Escherichia coli* activate NOD1-mediated immune responses in intestinal epithelial cells. Front Microbiol. 2018;9:498. 29616010 10.3389/fmicb.2018.00498 PMC5869251

[bib21] Caron F , du Chatelet IP, Leroy JP et al. From tailor-made to ready-to-wear meningococcal B vaccines: longitudinal study of a clonal meningococcal B outbreak. Lancet Infect Dis. 2011;11:455–63. 21489881 10.1016/S1473-3099(11)70027-5

[bib22] Caruana JC , Walper SA. Bacterial membrane vesicles and their applications as vaccines and in biotechnology. In: Kaparakis-Liaskos M, Kufer TA (eds). Bacterial Membrane Vesicles: Biogenesis, Functions and Applications. Cham: Springer International Publishing, 2020, 219–51.

[bib23] Chaddock AM , Mant A, Karnauchov I et al. A new type of signal peptide: central role of a twin-arginine motif in transfer signals for the delta pH-dependent thylakoidal protein translocase. EMBO J. 1995;14:2715–22. 7796800 10.1002/j.1460-2075.1995.tb07272.x PMC398390

[bib24] Chelakkot C , Choi Y, Kim DK et al. *Akkermansia muciniphila*-derived extracellular vesicles influence gut permeability through the regulation of tight junctions. Exp Mol Med. 2018;50:e450. 29472701 10.1038/emm.2017.282 PMC5903829

[bib25] Chen DJ , Osterrieder N, Metzger SM et al. Delivery of foreign antigens by engineered outer membrane vesicle vaccines. Proc Natl Acad Sci. 2010;107:3099–104. 20133740 10.1073/pnas.0805532107 PMC2840271

[bib26] Chen L , Valentine JL, Huang CJ et al. Outer membrane vesicles displaying engineered glycotopes elicit protective antibodies. Proc Natl Acad Sci. 2016;113:E3609–18. 27274048 10.1073/pnas.1518311113 PMC4932928

[bib27] Choi JW , Kim SC, Hong SH et al. Secretable small RNAs via outer membrane vesicles in periodontal pathogens. J Dent Res. 2017;96:458–66. 28068479 10.1177/0022034516685071

[bib28] Choi SJ , Kim MH, Jeon J et al. Active immunization with extracellular vesicles derived from *Staphylococcus aureus* effectively protects against Staphylococcal lung infections, mainly via Th1 cell-mediated immunity. PLoS One. 2015;10:e0136021. 26333035 10.1371/journal.pone.0136021 PMC4558092

[bib168_1623131936729] Chutkan H , Kuehn MJ. Context-Dependent Activation Kinetics Elicited by Soluble versus Outer Membrane Vesicle-Associated Heat-Labile Enterotoxin. Infect Immun. 2011;79:3760–9. 21708992 10.1128/IAI.05336-11 PMC3165487

[bib29] Cooke AC , Nello AV, Ernst RK et al. Analysis of *Pseudomonas aeruginosa* biofilm membrane vesicles supports multiple mechanisms of biogenesis. PLoS One. 2019;14:e0212275. 30763382 10.1371/journal.pone.0212275 PMC6375607

[bib30] Daleke-Schermerhorn MH , Felix T, Soprova Z et al. Decoration of outer membrane vesicles with multiple antigens by using an autotransporter approach. Appl Environ Microbiol. 2014;80:5854–65. 25038093 10.1128/AEM.01941-14 PMC4178611

[bib31] Davis JM , Carvalho HM, Rasmussen SB et al. Cytotoxic necrotizing factor type 1 delivered by outer membrane vesicles of uropathogenic *Escherichia coli* attenuates polymorphonuclear leukocyte antimicrobial activity and chemotaxis. Infect Immun. 2006;74:4401–8. 16861625 10.1128/IAI.00637-06 PMC1539604

[bib32] Durand V , Mackenzie J, de Leon J et al. Role of lipopolysaccharide in the induction of type I interferon-dependent cross-priming and IL-10 production in mice by meningococcal outer membrane vesicles. Vaccine. 2009;27:1912–22. 19368771 10.1016/j.vaccine.2009.01.109

[bib33] Elizagaray ML , Gomes MTR, Guimaraes ES et al. Canonical and non-canonical inflammasome activation by outer membrane vesicles derived from *Bordetella pertussis*. Front Immunol. 2020;11:1879. 32973778 10.3389/fimmu.2020.01879 PMC7468456

[bib34] Ellis TN , Leiman SA, Kuehn MJ. Naturally produced outer membrane vesicles from *Pseudomonas aeruginosa* elicit a potent innate immune response via combined sensing of both lipopolysaccharide and protein components. Infect Immun. 2010;78:3822–31. 20605984 10.1128/IAI.00433-10 PMC2937433

[bib35] Engevik MA , Danhof HA, Ruan W et al. *Fusobacterium nucleatum* secretes outer membrane vesicles and promotes intestinal inflammation. mBio. 2021;12:e02706–20. 33653893 10.1128/mBio.02706-20 PMC8092269

[bib36] Erwin AL , Smith AL. Nontypeable *Haemophilus influenzae*: understanding virulence and commensal behavior. Trends Microbiol. 2007;15:355–62. 17600718 10.1016/j.tim.2007.06.004

[bib37] Fantappie L , de Santis M, Chiarot E et al. Antibody-mediated immunity induced by engineered *Escherichia coli* OMVs carrying heterologous antigens in their lumen. J Extracell Vesicles. 2014;3:24015. 10.3402/jev.v3.24015 PMC4131003 25147647

[bib166_1623131247614] Fitzgerald KA , Kagan JC. Toll-like Receptors and the Control of Immunity. Cell. 2020;180:1044–66. 32164908 10.1016/j.cell.2020.02.041 PMC9358771

[bib38] Frasch CE , van Alphen L, Holst J et al. Outer membrane protein vesicle vaccines for meningococcal disease. Methods Mol Med. 2001;66:81–107. 21336749 10.1385/1-59259-148-5:81

[bib39] Fredriksen JH , Rosenqvist E, Wedege E et al. Production, characterization and control of MenB-vaccine “Folkehelsa”: an outer membrane vesicle vaccine against group B meningococcal disease. NIPH Ann. 1991;14:67–79. 1812438

[bib40] Frejd FY , Kim KT. Affibody molecules as engineered protein drugs. Exp Mol Med. 2017;49:e306. 28336959 10.1038/emm.2017.35 PMC5382565

[bib41] Fu-Hsiang SIDFT , Chih-Yun W, Shen-Long T. Decorating outer membrane vesicles with organophosphorus hydrolase and cellulose binding domain for organophosphate pesticide degradation. Chem Eng J. 2017;308:1–7.

[bib42] Gaspar EB , Prudencio CR, De Gaspari E. Experimental studies using OMV in a new platform of SARS-CoV-2 vaccines. Hum Vaccin Immunother. 2021;1–4., DOI: 10.1080/21645515.2021.1920272. 10.1080/21645515.2021.1920272 PMC8108191 33950776

[bib43] Gerke C , Colucci AM, Giannelli C et al. Production of a *Shigella sonnei* vaccine based on Generalized Modules for Membrane Antigens (GMMA), 1790GAHB. PLoS One. 2015;10:e0134478. 26248044 10.1371/journal.pone.0134478 PMC4527750

[bib44] Girardin SE , Boneca IG, Carneiro LA et al. Nod1 detects a unique muropeptide from Gram-negative bacterial peptidoglycan. Science. 2003;300:1584–7. 12791997 10.1126/science.1084677

[bib45] Gnopo YMD , Watkins HC, Stevenson TC et al. Designer outer membrane vesicles as immunomodulatory systems: reprogramming bacteria for vaccine delivery. Adv Drug Deliv Rev. 2017;114:132–42. 28501509 10.1016/j.addr.2017.05.003

[bib46] Grandi A , Tomasi M, Zanella I et al. Synergistic protective activity of tumor-specific epitopes engineered in bacterial outer membrane vesicles. Front Oncol. 2017;7:253. 29164053 10.3389/fonc.2017.00253 PMC5681935

[bib48] Guerrero-Mandujano A , Hernandez-Cortez C, Ibarra JA et al. The outer membrane vesicles: secretion system type zero. Traffic. 2017;18:425–32. 28421662 10.1111/tra.12488

[bib49] Gujrati V , Kim S, Kim SH et al. Bioengineered bacterial outer membrane vesicles as cell-specific drug-delivery vehicles for cancer therapy. ACS Nano. 2014;8:1525–37. 24410085 10.1021/nn405724x

[bib47] Gu TW , Wang MZ, Niu J et al. Outer membrane vesicles derived from *E. coli* as novel vehicles for transdermal and tumor targeting delivery. Nanoscale. 2020;12:18965–77. 32914815 10.1039/d0nr03698f

[bib50] Hagar JA , Powell DA, Aachoui Y et al. Cytoplasmic LPS activates caspase-11: implications in TLR4-independent endotoxic shock. Science. 2013;341:1250–3. 24031018 10.1126/science.1240988 PMC3931427

[bib51] Haurat MF , Aduse-Opoku J, Rangarajan M et al. Selective sorting of cargo proteins into bacterial membrane vesicles. J Biol Chem. 2011;286:1269–76. 21056982 10.1074/jbc.M110.185744 PMC3020734

[bib52] Hays MP , Houben D, Yang Y et al. Immunization with skp delivered on outer membrane vesicles protects mice against enterotoxigenic *Escherichia coli* challenge. Front Cell Infection Microbiol. 2018;8:132. 10.3389/fcimb.2018.00132 PMC5938412 29765911

[bib53] Holst J , Martin D, Arnold R et al. Properties and clinical performance of vaccines containing outer membrane vesicles from *Neisseria meningitidis*. Vaccine. 2009;27:B3–12. 19481313 10.1016/j.vaccine.2009.04.071

[bib54] Holst J , Oster P, Arnold R et al. Vaccines against meningococcal serogroup B disease containing outer membrane vesicles (OMV): lessons from past programs and implications for the future. Hum Vaccin Immunother. 2013;9:1241–53. 23857274 10.4161/hv.24129 PMC3901813

[bib55] Horstman AL , Kuehn MJ. Bacterial surface association of heat-labile enterotoxin through lipopolysaccharide after secretion via the general secretory pathway. J Biol Chem. 2002;277:32538–45. 12087095 10.1074/jbc.M203740200 PMC4391702

[bib56] Hoshino K , Takeuchi O, Kawai T et al. Cutting edge: Toll-like receptor 4 (TLR4)-deficient mice are hyporesponsive to lipopolysaccharide: evidence for TLR4 as the Lps gene product. J Immunol. 1999;162:3749–52. 10201887

[bib57] Howard CJ , Charleston B, Stephens SA et al. The role of dendritic cells in shaping the immune response. Anim Health Res Rev. 2004;5:191–5. 15984324 10.1079/ahr200468

[bib58] Imayoshi R , Cho T, Kaminishi H. NO production in RAW264 cells stimulated with *Porphyromonas gingivalis* extracellular vesicles. Oral Dis. 2011;17:83–9. 20646228 10.1111/j.1601-0825.2010.01708.x

[bib59] Intravacc . 2021a. https://www.intravacc.nl/ (27 June 2021, date last accessed).

[bib60] Intravacc . 2021b. https://www.intravacc.nl/news/intravacc-announces-positive-pre-clinical-data-intranasal-sars-cov-2-candidate-vaccine/(27 June 2021, date last accessed).

[bib61] Ismail S , Hampton MB, Keenan JI. *Helicobacter pylori* outer membrane vesicles modulate proliferation and interleukin-8 production by gastric epithelial cells. Infect Immun. 2003;71:5670–5. 14500487 10.1128/IAI.71.10.5670-5675.2003 PMC201067

[bib62] Jiang Y , Kong Q, Roland KL et al. Membrane vesicles of *Clostridium perfringens* type A strains induce innate and adaptive immunity. Int J Med Microbiol. 2014;304:431–43. 24631214 10.1016/j.ijmm.2014.02.006 PMC4285460

[bib63] Jin MS , Lee JO. Structures of TLR-ligand complexes. Curr Opin Immunol. 2008;20:414–9. 18585456 10.1016/j.coi.2008.06.002

[bib64] Jones EJ , Booth C, Fonseca S et al. The uptake, trafficking, and biodistribution of *Bacteroides thetaiotaomicron* generated outer membrane vesicles. Front Microbiol. 2020;11:57. 32117106 10.3389/fmicb.2020.00057 PMC7015872

[bib65] Kang CS , Ban M, Choi EJ et al. Extracellular vesicles derived from gut microbiota, especially *Akkermansia muciniphila*, protect the progression of dextran sulfate sodium-induced colitis. PLoS One. 2013;8:e76520. 24204633 10.1371/journal.pone.0076520 PMC3811976

[bib66] Kaparakis M , Turnbull L, Carneiro L et al. Bacterial membrane vesicles deliver peptidoglycan to NOD1 in epithelial cells. Cell Microbiol. 2010;12:372–85. 19888989 10.1111/j.1462-5822.2009.01404.x

[bib67] Kawai T , Akira S. The role of pattern-recognition receptors in innate immunity: update on Toll-like receptors. Nat Immunol. 2010;11:373–84. 20404851 10.1038/ni.1863

[bib68] Kayagaki N , Wong MT, Stowe IB et al. Noncanonical inflammasome activation by intracellular LPS independent of TLR4. Science. 2013;341:1246–9. 23887873 10.1126/science.1240248

[bib69] Keiser PB , Biggs-Cicatelli S, Moran EE et al. A phase 1 study of a meningococcal native outer membrane vesicle vaccine made from a group B strain with deleted *lpxL1* and *synX*, over-expressed factor H binding protein, two PorAs and stabilized OpcA expression. Vaccine. 2011;29:1413–20. 21199704 10.1016/j.vaccine.2010.12.039

[bib70] Keiser PB , Gibbs BT, Coster TS et al. A phase 1 study of a group B meningococcal native outer membrane vesicle vaccine made from a strain with deleted *lpxL2* and *synX* and stable expression of opcA. Vaccine. 2010;28:6970–6. 20732470 10.1016/j.vaccine.2010.08.048

[bib71] Kesty NC , Kuehn MJ. Incorporation of heterologous outer membrane and periplasmic proteins into *Escherichia coli* outer membrane vesicles. J Biol Chem. 2004;279:2069–76. 14578354 10.1074/jbc.M307628200 PMC3525464

[bib72] Kim JY , Doody AM, Chen DJ et al. Engineered bacterial outer membrane vesicles with enhanced functionality. J Mol Biol. 2008;380:51–66. 18511069 10.1016/j.jmb.2008.03.076 PMC4617544

[bib73] Kim OY , Dinh NT, Park HT et al. Bacterial protoplast-derived nanovesicles for tumor targeted delivery of chemotherapeutics. Biomaterials. 2017;113:68–79. 27810643 10.1016/j.biomaterials.2016.10.037

[bib74] Kim OY , Park HT, Dinh NTH et al. Bacterial outer membrane vesicles suppress tumor by interferon-gamma-mediated antitumor response. Nat Commun. 2017;8:626. 28931823 10.1038/s41467-017-00729-8 PMC5606984

[bib75] Koeppen K , Hampton TH, Jarek M et al. A novel mechanism of host–pathogen interaction through sRNA in bacterial outer membrane vesicles. PLoS Pathog. 2016;12:e1005672. 27295279 10.1371/journal.ppat.1005672 PMC4905634

[bib76] Kraft JC , Freeling JP, Wang Z et al. Emerging research and clinical development trends of liposome and lipid nanoparticle drug delivery systems. J Pharm Sci. 2014;103:29–52. 24338748 10.1002/jps.23773 PMC4074410

[bib77] Kuerban K , Gao X, Zhang H et al. Doxorubicin-loaded bacterial outer-membrane vesicles exert enhanced anti-tumor efficacy in non-small-cell lung cancer. Acta Pharmaceutica Sinica B. 2020;10:1534–48. 32963948 10.1016/j.apsb.2020.02.002 PMC7488491

[bib78] Kuipers K , Daleke-Schermerhorn MH, Jong WS et al. Salmonella outer membrane vesicles displaying high densities of pneumococcal antigen at the surface offer protection against colonization. Vaccine. 2015;33:2022–9. 25776921 10.1016/j.vaccine.2015.03.010

[bib80] Kulkarni HM , Nagaraj R, Jagannadham MV. Protective role of *E. coli* outer membrane vesicles against antibiotics. Microbiol Res. 2015;181:1–7. 26640046 10.1016/j.micres.2015.07.008

[bib79] Kulkarni HM , Jagannadham MV. Biogenesis and multifaceted roles of outer membrane vesicles from Gram-negative bacteria. Microbiology. 2014;160:2109–21. 25069453 10.1099/mic.0.079400-0

[bib81] Lapinet JA , Scapini P, Calzetti F et al. Gene expression and production of tumor necrosis factor alpha, interleukin-1 beta (IL-1β), IL-8, macrophage inflammatory protein 1 alpha (MIP-1α), MIP-1β, and gamma interferon-inducible protein 10 by human neutrophils stimulated with group B meningococcal outer membrane vesicles. Infect Immun. 2000;68:6917–23. 11083814 10.1128/iai.68.12.6917-6923.2000 PMC97799

[bib82] Lappann M , Otto A, Becher D et al. Comparative proteome analysis of spontaneous outer membrane vesicles and purified outer membranes of *Neisseria meningitidis*. J Bacteriol. 2013;195:4425–35. 23893116 10.1128/JB.00625-13 PMC3807460

[bib83] Launay O , Lewis DJM, Anemona A et al. Safety profile and immunologic responses of a novel vaccine against *Shigella sonnei* administered intramuscularly, intradermally and intranasally: results from two parallel randomized phase 1 clinical studies in healthy adult volunteers in Europe. EBioMedicine. 2017;22:164–72. 28735965 10.1016/j.ebiom.2017.07.013 PMC5552227

[bib84] Leduc I , Connolly KL, Begum A et al. The serogroup B meningococcal outer membrane vesicle-based vaccine 4CMenB induces cross-species protection against *Neisseria gonorrhoeae*. PLoS Pathog. 2020;16:e1008602. 33290434 10.1371/journal.ppat.1008602 PMC7748408

[bib85] Lee SR , Kim SH, Jeong KJ et al. Multi-immunogenic outer membrane vesicles derived from an MsbB-deficient *Salmonella enterica* serovar typhimurium mutant. J Microbiol Biotechnol. 2009;19:1271–9. 19884791

[bib87] Leitner DR , Lichtenegger S, Temel P et al. A combined vaccine approach against *Vibrio cholerae* and ETEC based on outer membrane vesicles. Front Microbiol. 2015;6:823. 26322032 10.3389/fmicb.2015.00823 PMC4531250

[bib86] Leitner DR , Feichter S, Schild-Prufert K et al. Lipopolysaccharide modifications of a cholera vaccine candidate based on outer membrane vesicles reduce endotoxicity and reveal the major protective antigen. Infect Immun. 2013;81:2379–93. 23630951 10.1128/IAI.01382-12 PMC3697601

[bib88] Li Q , Ren J, Xian H et al. rOmpF and OMVs as efficient subunit vaccines against *Salmonella enterica* serovar Enteritidis infections in poultry farms. Vaccine. 2020a;38:7094–9. 32951940 10.1016/j.vaccine.2020.08.074

[bib89] Li R , Liu Q. Engineered bacterial outer membrane vesicles as multifunctional delivery platforms. Front Mater. 2020b;7, DOI: 10.3389/fmats.2020.00202.

[bib90] Li S , Chen DQ, Ji L et al. Development of different methods for preparing *Acinetobacter baumannii* outer membrane vesicles vaccine: impact of preparation method on protective efficacy. Front Immunol. 2020c;11:1069. 32655550 10.3389/fimmu.2020.01069 PMC7324643

[bib92] Liu Y , Defourny KAY, Smid EJ et al. Gram-positive bacterial extracellular vesicles and their impact on health and disease. Front Microbiol. 2018;9:1502. 30038605 10.3389/fmicb.2018.01502 PMC6046439

[bib91] Li Y , Zhao R, Cheng K et al. Bacterial outer membrane vesicles presenting programmed death 1 for improved cancer immunotherapy via immune activation and checkpoint inhibition. ACS Nano. 2020d;14:16698–711. 33232124 10.1021/acsnano.0c03776

[bib93] Mahla RS , Reddy MC, Prasad DV et al. Sweeten PAMPs: role of sugar complexed PAMPs in innate immunity and vaccine biology. Front Immunol. 2013;4:248. 24032031 10.3389/fimmu.2013.00248 PMC3759294

[bib94] McBroom AJ , Johnson AP, Vemulapalli S et al. Outer membrane vesicle production by *Escherichia coli* is independent of membrane instability. J Bacteriol. 2006;188:5385–92. 16855227 10.1128/JB.00498-06 PMC1540050

[bib95] Micoli F , MacLennan CA. Outer membrane vesicle vaccines. Semin Immunol. 2020;50:101433. 33309166 10.1016/j.smim.2020.101433

[bib96] Mirlashari MR , Hoiby EA, Holst J et al. Outer membrane vesicles from *Neisseria meningitidis*: effects on tissue factor and plasminogen activator inhibitor-2 production in human monocytes. Thromb Res. 2001;102:375–80. 11369430 10.1016/s0049-3848(01)00256-0

[bib97] Mitra S , Chakrabarti MK, Koley H. Multi-serotype outer membrane vesicles of Shigellae confer passive protection to the neonatal mice against shigellosis. Vaccine. 2013;31:3163–73. 23684822 10.1016/j.vaccine.2013.05.001

[bib98] Muralinath M , Kuehn MJ, Roland KL et al. Immunization with *Salmonella enterica* serovar Typhimurium-derived outer membrane vesicles delivering the pneumococcal protein PspA confers protection against challenge with *Streptococcus pneumoniae*. Infect Immun. 2011;79:887–94. 21115718 10.1128/IAI.00950-10 PMC3028854

[bib99] Nevot M , Deroncele V, Messner P et al. Characterization of outer membrane vesicles released by the psychrotolerant bacterium *Pseudoalteromonas antarctica* NF3. Environ Microbiol. 2006;8:1523–33. 16913913 10.1111/j.1462-2920.2006.01043.x PMC4379500

[bib100] O'Donoghue EJ , Krachler AM. Mechanisms of outer membrane vesicle entry into host cells. Cell Microbiol. 2016;18:1508–17. 27529760 10.1111/cmi.12655 PMC5091637

[bib101] Obiero CW , Ndiaye AGW, Scire AS et al. A phase 2a randomized study to evaluate the safety and immunogenicity of the 1790GAHB generalized modules for membrane antigen vaccine against *Shigella sonnei* administered intramuscularly to adults from a Shigellosis-endemic country. Front Immunol. 2017;8:1884. 29375556 10.3389/fimmu.2017.01884 PMC5763125

[bib102] Olaya-Abril A , Prados-Rosales R, McConnell MJ et al. Characterization of protective extracellular membrane-derived vesicles produced by *Streptococcus pneumoniae*. J Proteomics. 2014;106:46–60. 24769240 10.1016/j.jprot.2014.04.023

[bib103] Oliver DB , Beckwith J. Regulation of a membrane component required for protein secretion in *Escherichia coli*. Cell. 1982;30:311–9. 6751561 10.1016/0092-8674(82)90037-x

[bib104] Olofsson A , Vallstrom A, Petzold K et al. Biochemical and functional characterization of *Helicobacter pylori* vesicles. Mol Microbiol. 2010;77:1539–55. 20659286 10.1111/j.1365-2958.2010.07307.x PMC3068288

[bib105] Oster P , Lennon D, O'Hallahan J et al. MeNZB: a safe and highly immunogenic tailor-made vaccine against the New Zealand *Neisseria meningitidis* serogroup B disease epidemic strain. Vaccine. 2005;23:2191–6. 15755593 10.1016/j.vaccine.2005.01.063

[bib106] Park BS , Song DH, Kim HM et al. The structural basis of lipopolysaccharide recognition by the TLR4-MD-2 complex. Nature. 2009;458:1191–5. 19252480 10.1038/nature07830

[bib107] Park M , Sun Q, Liu F et al. Positional assembly of enzymes on bacterial outer membrane vesicles for cascade reactions. PLoS One. 2014;9:e97103. 24820175 10.1371/journal.pone.0097103 PMC4018249

[bib109] Petousis-Harris H , Paynter J, Morgan J et al. Effectiveness of a group B outer membrane vesicle meningococcal vaccine against gonorrhoea in New Zealand: a retrospective case-control study. Lancet North Am Ed. 2017;390:1603–10. 10.1016/S0140-6736(17)31449-6 28705462

[bib108] Petousis-Harris H . Impact of meningococcal group B OMV vaccines, beyond their brief. Hum Vaccin Immunother. 2018;14:1058–63. 29048985 10.1080/21645515.2017.1381810 PMC5989908

[bib110] Philpott DJ , Sorbara MT, Robertson SJ et al. NOD proteins: regulators of inflammation in health and disease. Nat Rev Immunol. 2014;14:9–23. 24336102 10.1038/nri3565

[bib111] Pizza M , Bekkat-Berkani R, Rappuoli R. Vaccines against Meningococcal diseases. Microorganisms. 2020;8:1521. 33022961 10.3390/microorganisms8101521 PMC7601370

[bib112] Poltorak A , He X, Smirnova I et al. Defective LPS signaling in C3H/HeJ and C57BL/10ScCr mice: mutations in Tlr4 gene. Science. 1998;282:2085–8. 9851930 10.1126/science.282.5396.2085

[bib113] Price NL , Goyette-Desjardins G, Nothaft H et al. Glycoengineered outer membrane vesicles: a novel platform for bacterial vaccines. Sci Rep. 2016;6:24931. 27103188 10.1038/srep24931 PMC4840304

[bib114] Rappazzo CG , Watkins HC, Guarino CM et al. Recombinant M2e outer membrane vesicle vaccines protect against lethal influenza A challenge in BALB/c mice. Vaccine. 2016;34:1252–8. 26827663 10.1016/j.vaccine.2016.01.028

[bib115] Rasti ES , Schappert ML, Brown AC. Association of *Vibrio cholerae* 569B outer membrane vesicles with host cells occurs in a GM1-independent manner. Cell Microbiol. 2018;20:e12828. 29377560 10.1111/cmi.12828 PMC5980675

[bib116] Rehman A , Sina C, Gavrilova O et al. Nod2 is essential for temporal development of intestinal microbial communities. Gut. 2011;60:1354–62. 21421666 10.1136/gut.2010.216259

[bib117] Reyes-Robles T , Dillard RS, Cairns LS et al. *Vibrio cholerae* outer membrane vesicles inhibit bacteriophage infection. J Bacteriol. 2018;200:e00792–17. 29661863 10.1128/JB.00792-17 PMC6040182

[bib118] Rivera J , Cordero RJ, Nakouzi AS et al. *Bacillus anthracis* produces membrane-derived vesicles containing biologically active toxins. Proc Natl Acad Sci. 2010;107:19002–7. 20956325 10.1073/pnas.1008843107 PMC2973860

[bib119] Roier S , Leitner DR, Iwashkiw J et al. Intranasal immunization with nontypeable *Haemophilus influenzae* outer membrane vesicles induces cross-protective immunity in mice. PLoS One. 2012;7:e42664. 22880074 10.1371/journal.pone.0042664 PMC3411803

[bib120] Rossi O , Caboni M, Negrea A et al. Toll-like receptor activation by generalized modules for membrane antigens from lipid A mutants of *Salmonella enterica* serovars Typhimurium and Enteritidis. Clin Vaccine Immunol. 2016;23:304–14. 26865597 10.1128/CVI.00023-16 PMC4820502

[bib121] Rossi O , Citiulo F, Mancini F. Outer membrane vesicles: moving within the intricate labyrinth of assays that can predict risks of reactogenicity in humans. Hum Vaccin Immunother. 2021;17:601–13. 32687736 10.1080/21645515.2020.1780092 PMC7899674

[bib122] Rossi O , Pesce I, Giannelli C et al. Modulation of endotoxicity of Shigella generalized modules for membrane antigens (GMMA) by genetic lipid A modifications: relative activation of TLR4 and TLR2 pathways in different mutants. J Biol Chem. 2014;289:24922–35. 25023285 10.1074/jbc.M114.566570 PMC4155660

[bib123] Sada E , Katoh S, Terashima M et al. Reaction properties of hydrophobic enzymes and their immobilization on phospholipid composite membranes. Biotechnol Bioeng. 1987;30:117–22. 18576591 10.1002/bit.260300117

[bib124] Salvachua D , Werner AZ, Pardo I et al. Outer membrane vesicles catabolize lignin-derived aromatic compounds in *Pseudomonas putida* KT2440. Proc Natl Acad Sci. 2020;117:9302–10. 32245809 10.1073/pnas.1921073117 PMC7196908

[bib125] Salverda ML , Meinderts SM, Hamstra HJ et al. Surface display of a borrelial lipoprotein on meningococcal outer membrane vesicles. Vaccine. 2016;34:1025–33. 26801064 10.1016/j.vaccine.2016.01.019

[bib126] Schaar V , de Vries SP, Perez Vidakovics ML et al. Multicomponent *Moraxella catarrhalis* outer membrane vesicles induce an inflammatory response and are internalized by human epithelial cells. Cell Microbiol. 2011;13:432–49. 21044239 10.1111/j.1462-5822.2010.01546.x

[bib127] Schetters STT , Jong WSP, Horrevorts SK et al. Outer membrane vesicles engineered to express membrane-bound antigen program dendritic cells for cross-presentation to CD8(+) T cells. Acta Biomater. 2019;91:248–57. 31003032 10.1016/j.actbio.2019.04.033

[bib128] Schroeder J , Aebischer T. Recombinant outer membrane vesicles to augment antigen-specific live vaccine responses. Vaccine. 2009;27:6748–54. 19748581 10.1016/j.vaccine.2009.08.106

[bib129] Schultz H , Hume J, Zhang DS et al. A novel role for the bactericidal/permeability increasing protein in interactions of Gram-negative bacterial outer membrane blebs with dendritic cells. J Immunol. 2007;179:2477–84. 17675509 10.4049/jimmunol.179.4.2477

[bib131] Schwechheimer C , Kuehn MJ. Outer-membrane vesicles from Gram-negative bacteria: biogenesis and functions. Nat Rev Microbiol. 2015;13:605–19. 26373371 10.1038/nrmicro3525 PMC5308417

[bib130] Schwechheimer C , Kuehn MJ. Synthetic effect between envelope stress and lack of outer membrane vesicle production in *Escherichia coli*. J Bacteriol. 2013;195:4161–73. 23852867 10.1128/JB.02192-12 PMC3754735

[bib132] Schwechheimer C , Kulp A, Kuehn MJ. Modulation of bacterial outer membrane vesicle production by envelope structure and content. BMC Microbiol. 2014;14:324. 25528573 10.1186/s12866-014-0324-1 PMC4302634

[bib133] Semchenko EA , Tan A, Borrow R et al. The serogroup B meningococcal vaccine Bexsero elicits antibodies to *Neisseria gonorrhoeae*. Clin Infect Dis. 2019;69:1101–11. 30551148 10.1093/cid/ciy1061 PMC6743822

[bib134] Serruto D , Bottomley MJ, Ram S et al. The new multicomponent vaccine against meningococcal serogroup B, 4CMenB: immunological, functional and structural characterization of the antigens. Vaccine. 2012;30:B87–97. 22607904 10.1016/j.vaccine.2012.01.033 PMC3360877

[bib135] Sevestre J , Hong E, Delbos V et al. Durability of immunogenicity and strain coverage of MenBvac, a meningococcal vaccine based on outer membrane vesicles: lessons of the Normandy campaign. Vaccine. 2017;35:4029–33. 28624305 10.1016/j.vaccine.2017.05.065

[bib136] Shehata MM , Mostafa A, Teubner L et al. Bacterial outer membrane vesicles (OMVs)-based dual vaccine for influenza A H1N1 virus and MERS-CoV. Vaccines. 2019;7:46. 31141982 10.3390/vaccines7020046 PMC6631769

[bib137] Shen Y , Giardino Torchia ML, Lawson GW et al. Outer membrane vesicles of a human commensal mediate immune regulation and disease protection. Cell Host Microbe. 2012;12:509–20. 22999859 10.1016/j.chom.2012.08.004 PMC3895402

[bib138] Soderblom T , Oxhamre C, Wai SN et al. Effects of the *Escherichia coli* toxin cytolysin A on mucosal immunostimulation via epithelial Ca^2+^ signalling and Toll-like receptor 4. Cell Microbiol. 2005;7:779–88. 15888081 10.1111/j.1462-5822.2005.00510.x

[bib139] Song T , Mika F, Lindmark B et al. A new *Vibrio cholerae* sRNA modulates colonization and affects release of outer membrane vesicles. Mol Microbiol. 2008;70:100–11. 18681937 10.1111/j.1365-2958.2008.06392.x PMC2628432

[bib140] Sonnenborn U . *Escherichia coli* strain Nissle 1917-from bench to bedside and back: history of a special *Escherichia coli* strain with probiotic properties. FEMS Microbiol Lett. 2016;363:fnw212. 27619890 10.1093/femsle/fnw212

[bib141] Stentz R , Osborne S, Horn N et al. A bacterial homolog of a eukaryotic inositol phosphate signaling enzyme mediates cross-kingdom dialog in the mammalian gut. Cell Rep. 2014;6:646–56. 24529702 10.1016/j.celrep.2014.01.021 PMC3969271

[bib142] Tavano R , Franzoso S, Cecchini P et al. The membrane expression of *Neisseria meningitidis* adhesin A (NadA) increases the proimmune effects of MenB OMVs on human macrophages, compared with NadA- OMVs, without further stimulating their proinflammatory activity on circulating monocytes. J Leukoc Biol. 2009;86:143–53. 19401383 10.1189/jlb.0109030

[bib143] Thompson SS , Naidu YM, Pestka JJ. Ultrastructural localization of an extracellular protease in *Pseudomonas fragi* by using the peroxidase-antiperoxidase reaction. Appl Environ Microbiol. 1985;50:1038–42. 3909961 10.1128/aem.50.4.1038-1042.1985 PMC291789

[bib144] Tinsley CR , Nassif X. Analysis of the genetic differences between *Neisseria meningitidis* and *Neisseria gonorrhoeae*: two closely related bacteria expressing two different pathogenicities. Proc Natl Acad Sci. 1996;93:11109–14. 8855317 10.1073/pnas.93.20.11109 PMC38292

[bib145] Tunheim G , Arnemo M, Naess LM et al. Preclinical immunogenicity and functional activity studies of an A+W meningococcal outer membrane vesicle (OMV) vaccine and comparisons with existing meningococcal conjugate- and polysaccharide vaccines. Vaccine. 2013;31:6097–106. 24120679 10.1016/j.vaccine.2013.09.044

[bib146] Valentine JL , Chen L, Perregaux EC et al. Immunization with outer membrane vesicles displaying designer glycotopes yields class-switched, glycan-specific antibodies. Cell Chem Biol. 2016;23:655–65. 27341433 10.1016/j.chembiol.2016.05.014 PMC5116915

[bib153] Vanaja SK , Russo AJ, Behl B et al. Bacterial outer membrane vesicles mediate cytosolic localization of LPS and caspase-11 activation. Cell. 2016;165:1106–19. 27156449 10.1016/j.cell.2016.04.015 PMC4874922

[bib147] van Alphen L , Caugant DA, Duim B et al. Differences in genetic diversity of nonencapsulated *Haemophilus influenzae* from various diseases. Microbiology. 1997;143:1423–31. 9141705 10.1099/00221287-143-4-1423

[bib151] van der Ley P , Steeghs L, Hamstra HJ et al. Modification of lipid A biosynthesis in *Neisseria meningitidis lpxL* mutants: influence on lipopolysaccharide structure, toxicity, and adjuvant activity. Infect Immun. 2001;69:5981–90. 11553534 10.1128/IAI.69.10.5981-5990.2001 PMC98725

[bib152] van der Pol L , Stork M, van der Ley P. Outer membrane vesicles as platform vaccine technology. Biotechnol J. 2015;10:1689–706. 26912077 10.1002/biot.201400395 PMC4768646

[bib148] van de Waterbeemd B , Mommen GP, Pennings JL et al. Quantitative proteomics reveals distinct differences in the protein content of outer membrane vesicle vaccines. J Proteome Res. 2013;12:1898–908. 23410224 10.1021/pr301208g

[bib149] van de Waterbeemd B , Streefland M, van der Ley P et al. Improved OMV vaccine against *Neisseria meningitidis* using genetically engineered strains and a detergent-free purification process. Vaccine. 2010;28:4810–6. 20483197 10.1016/j.vaccine.2010.04.082

[bib150] van de Waterbeemd B , Zomer G, Kaaijk P et al. Improved production process for native outer membrane vesicle vaccine against *Neisseria meningitidis*. PLoS One. 2013b;8:e65157. 23741478 10.1371/journal.pone.0065157 PMC3669287

[bib154] Vasilyeva NV , Tsfasman IM, Suzina NE et al. Secretion of bacteriolytic endopeptidase L5 of *Lysobacter*sp. XL1 into the medium by means of outer membrane vesicles. FEBS J. 2008;275:3827–35. 18573103 10.1111/j.1742-4658.2008.06530.x

[bib155] Vidakovics ML , Jendholm J, Morgelin M et al. B cell activation by outer membrane vesicles: a novel virulence mechanism. PLoS Pathog. 2010;6:e1000724. 20090836 10.1371/journal.ppat.1000724 PMC2799554

[bib156] Wai SN , Takade A, Amako K. The release of outer membrane vesicles from the strains of enterotoxigenic *Escherichia coli*. Microbiol Immunol. 1995;39:451–6. 8569529 10.1111/j.1348-0421.1995.tb02228.x

[bib157] Wang S , Huang W, Li K et al. Engineered outer membrane vesicle is potent to elicit HPV16E7-specific cellular immunity in a mouse model of TC-1 graft tumor. Int J Nanomed. 2017;12:6813–25. 10.2147/IJN.S143264 PMC5602458 28979120

[bib158] Wang X , Thompson CD, Weidenmaier C et al. Release of *Staphylococcus aureus* extracellular vesicles and their application as a vaccine platform. Nat Commun. 2018;9:1379. 29643357 10.1038/s41467-018-03847-z PMC5895597

[bib159] Winter J , Letley D, Rhead J et al. *Helicobacter pylori* membrane vesicles stimulate innate pro- and anti-inflammatory responses and induce apoptosis in Jurkat T cells. Infect Immun. 2014;82:1372–81. 24421041 10.1128/IAI.01443-13 PMC3993389

[bib167_1623131545916] Yarovinsky F , Zhang D, Andersen JF et al. TLR11 Activation of Dendritic Cells by a Protozoan Profilin-Like Protein. Science. 2005;308:1626–9. 15860593 10.1126/science.1109893

[bib160] Yu H , Kim KS. YgfZ contributes to secretion of cytotoxic necrotizing factor 1 into outer-membrane vesicles in *Escherichia coli*. Microbiology. 2012;158:612–21. 22174383 10.1099/mic.0.054122-0 PMC3352114

[bib161] Zamyatina A , Heine H. Lipopolysaccharide recognition in the crossroads of TLR4 and caspase-4/11 mediated inflammatory pathways. Front Immunol. 2020;11:585146. 33329561 10.3389/fimmu.2020.585146 PMC7732686

[bib162] Zariri A , Beskers J, van de Waterbeemd B et al. Meningococcal outer membrane vesicle composition-dependent activation of the innate immune response. Infect Immun. 2016;84:3024–33. 27481244 10.1128/IAI.00635-16 PMC5038073

[bib163] Zhang Y , Fang Z, Li R et al. Design of outer membrane vesicles as cancer vaccines: a new toolkit for cancer therapy. Cancers. 2019;11:1314. 31500086 10.3390/cancers11091314 PMC6769604

[bib164] Zhao K , Deng X, He C et al. *Pseudomonas aeruginosa* outer membrane vesicles modulate host immune responses by targeting the Toll-like receptor 4 signaling pathway. Infect Immun. 2013;81:4509–18. 24082079 10.1128/IAI.01008-13 PMC3837971

[bib165] Zingl FG , Kohl P, Cakar F et al. Outer membrane vesiculation facilitates surface exchange and *in vivo* adaptation of *Vibrio cholerae*. Cell Host Microbe. 2020;27:225–237.e8. 31901519 10.1016/j.chom.2019.12.002 PMC7155939

